# Local Geometric Perception for Climbing Robots on Double-Deck Steel Truss Bridges Using CAD Hole-Layout Priors and Depth-Derived Geometry

**DOI:** 10.3390/s26185855

**Published:** 2026-09-16

**Authors:** Jie Hou, Hongbing Zhang, Jiahao Lin, Xiaoyu Li, Xiaochen Wei, Yaoyu Zhu, Zhonghong Dong

**Affiliations:** 1 School of Mechanical Engineering and Intelligent Equipment, Chang’an University, Xi’an 710064, China; 2 CCCC Highway Bridge National Engineering Research Centre Co., Ltd., Beijing 100011, China

**Keywords:** double-deck steel truss bridges, robotic inspection, CAD structural priors, multilayer 2.5D elevation representation, depth sensing, local geometric perception

## Abstract

Close-range bridge inspection exposes personnel to risks associated with work at height and in confined spaces. Magnetic climbing robots can reduce this exposure, yet dense fastener groups and unsupported regions beyond truss member edges impose local geometric constraints on robot motion. Forward-looking depth observations of these regions are often partial and noisy. This study presents a local geometric perception method that produces two constraints. A computer-aided design (CAD)-constrained branch matches partial rivet observations to hole-layout templates and converts the recovered layout into an inward-offset rivet-passage boundary. A CAD-independent depth branch fits the supporting-surface boundary near a truss member edge. The method was evaluated in simulation, laboratory mock-ups, and a single-case field feasibility study. Under simulation conditions, CAD-constrained recovery reduced lateral mean absolute error by 83.17–85.14% relative to a geometry-only ablation baseline, and all generated passage boundaries retained positive theoretical clearance from the corresponding fastener contours. Laboratory trials achieved template locking; qualitative overlay inspection found no visible boundary intrusion for three rivet layouts. The single-case field study also achieved template locking and generated a passage boundary without visible intrusion into the fastener contours using a manually measured bolt-layout prior. The member-edge branch produced valid fits in 79.59% of field frames, with a mean branch processing time of 23.37 ms. These results support the feasibility of combining CAD hole-layout priors and depth geometry to provide complementary local geometric constraints for subsequent motion planning.

## 1. Introduction

River- and sea-crossing bridges are critical links in regional transport networks [1]. Double-deck steel truss bridges carry road and rail traffic on separate levels, combining high transport capacity with efficient use of structural space [2]. Their long-term service requires repeated close-range inspection of coatings, welds, and fastened connections [3,4,5]. Conventional inspection exposes personnel to work at height and in confined spaces across extensive structural areas, and defect localization may vary among inspectors [6]. Climbing robots can reduce personnel exposure and provide a repeatable platform for close-range data acquisition.

Continuous robotic inspection must address both unobstructed steel surfaces and geometrically constrained regions. This study uses a wheeled magnetic climbing robot for close-range bridge inspection (Section 2.1) and focuses on two such constraints: joints with dense fasteners and truss member edges. Joint regions contain gusset plates and dense groups of protruding fasteners that form regular, repetitive row-and-column patterns [7]. In some joint configurations, comparatively wide gaps between adjacent fastener columns may provide the geometric basis for candidate passage regions for compact robots. At a truss member edge, the supporting steel surface terminates, and movement into the unsupported region beyond the edge can reduce wheel support and magnetic adhesion, creating a fall hazard. Because the target bridge primarily uses riveted joints, the regularly arranged protruding fasteners are termed rivets throughout the method.

Environmental perception supplies the local geometry required for motion decisions and inspection tasks [8]. Previous bridge-climbing studies have focused mainly on mechanism design, motion control, teleoperation, and inspection data acquisition [9,10,11,12,13,14]. Some systems have also used prescribed-path tracking or offline planning: Song et al. [15] demonstrated path tracking inside a comparatively regular steel box girder, and Xia et al. [16] generated full-coverage paths from an offline map derived from building information modeling (BIM). These approaches improve robotic access but remain dependent on operators, predefined paths, or offline environmental information. A recent review likewise identified environmental perception and deployment beyond laboratory settings as persistent challenges for mobile construction robots [17].

Perception methods developed for other climbing robots offer useful but incomplete precedents. Li et al. [18] extracted weld seams from spherical tanks for inspection-path generation. Zhu et al. [19] combined odometry with structural landmarks for state estimation and parametric mapping in truss environments. Gu et al. [20] imposed surface-continuity constraints on state estimation over weak-texture free-form surfaces, whereas Teixeira et al. [21] used online mapping to predict inspectable regions on initially unknown tank surfaces. These methods address localization, mapping, or task geometry, but they do not directly resolve the paired local constraints posed by dense rivet groups and supporting-surface boundaries on double-deck steel truss bridges.

Digital structural models can provide geometric and semantic priors for structured environments. CAD- and BIM-assisted methods have used such information for robot localization, prior-map construction, semantic modeling, observation association, and as-built model updating [22,23,24,25,26]. Most of these applications treat the digital model as a localization or information management resource. The potential of digital structural information to support autonomous environmental perception by climbing robots on double-deck steel truss bridges remains insufficiently explored.

To address the local perception requirements of climbing robots on double-deck steel truss bridges, this study proposes a local geometric perception method that combines CAD hole-layout priors with online depth geometry. The main contributions are threefold. First, the method establishes a two-branch perception framework for two recurring constrained regions in this bridge setting: a rivet-group branch for joint regions with dense rivets and a member-edge branch for supporting-surface termination at truss member edges. Each branch produces a separate local geometric constraint for its target region. Second, a multilayer 2.5D representation serves as the shared geometric front end. The front end uses relative-height analysis, connected-component analysis, and geometric screening to identify rivet candidates; it uses height and valid-depth cues to extract member-edge points. Third, the rivet-group branch uses predefined CAD hole-layout relationships to constrain rivet-center estimates obtained from noisy or incomplete depth observations and recover a consistent group-level layout. It then projects theoretical rivet contours from the recovered layout and constructs an inward-offset candidate passage boundary. The member-edge branch fits the extracted edge points to convert discrete observations into a continuous supporting-surface boundary. Simulation quantitatively evaluates rivet localization, rivet-boundary clearance, and member-edge fitting. Laboratory tests and a single-case field study assess functional feasibility using real depth observations.

The remainder of this paper is organized as follows. Section 2 describes the robot-dependent geometric requirements and CAD hole-layout templates. Section 3 presents the multilayer 2.5D representation, rivet-group recovery, rivet-passage boundary construction, member-edge fitting, and exploratory candidate screening. Section 4 reports simulation, laboratory, and field evaluations. Section 5 discusses the evidence and limitations, and Section 6 concludes this study.

## 2. Robotic System and CAD Structural Priors

### 2.1. Robot Configuration and Geometric Requirements

**Figure 1:**
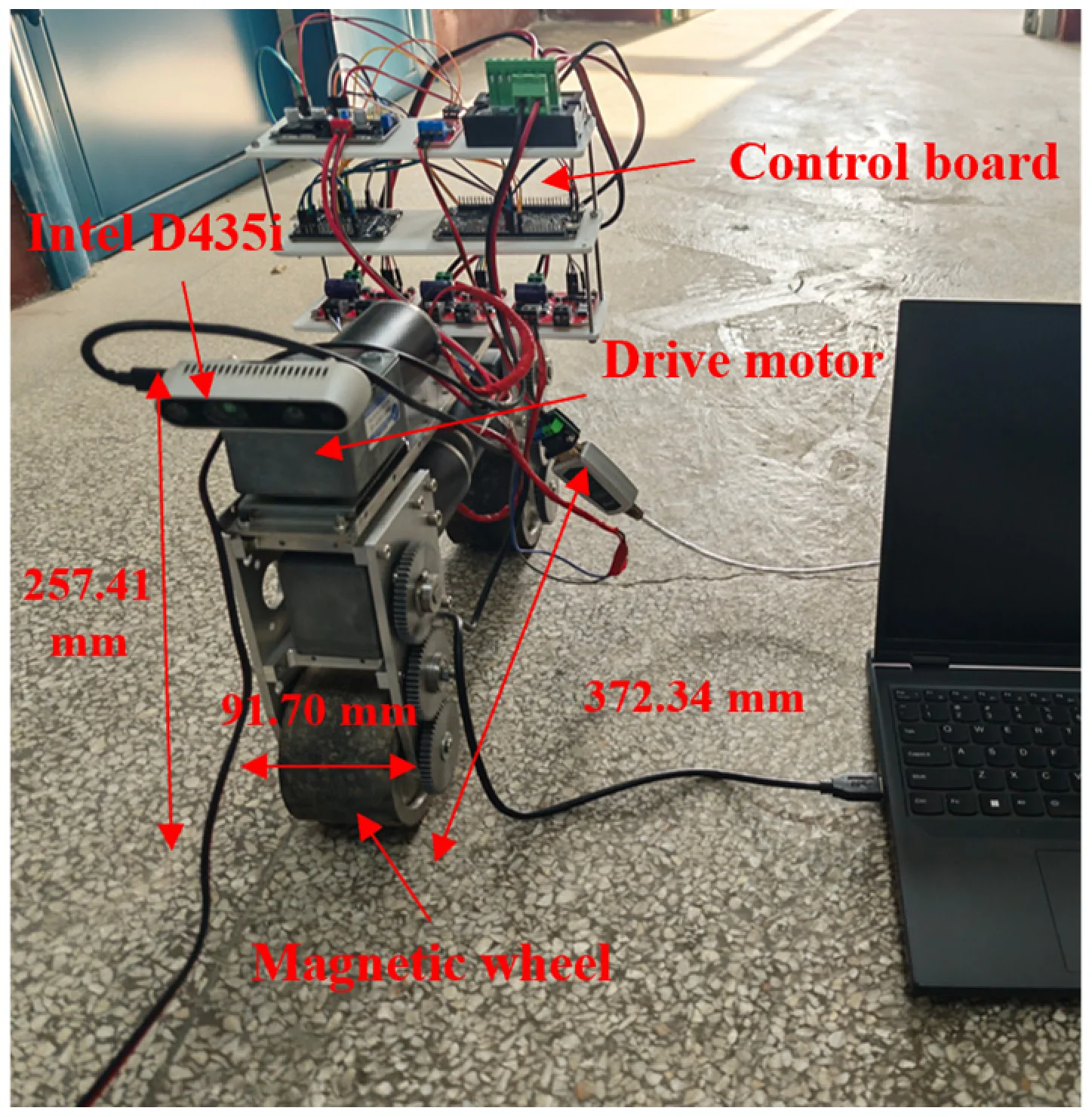
Overall configuration of the wheeled magnetic climbing inspection robot.

Figure 1 shows the wheeled magnetic climbing robot developed for the target bridge. Its front and rear magnetic wheel modules are arranged in tandem along the travel direction to reduce the transverse envelope for candidate gaps between rivet columns. The magnetic wheels are intended to provide adhesion on the steel surface; the present study does not evaluate adhesion safety.

The robot geometry establishes two local perception requirements. The permanent magnet arrangement and magnetic-yoke thickness affect adhesion and load capacity [27], whereas the wheel modules and adjacent components define the transverse envelope that a passage must accommodate. Near this limit, error in the estimated rivet centers or outer contours can overestimate the available width. The minimum passage width is a fixed screening parameter derived from the robot transverse envelope, not a demonstrated traversal clearance. A member edge imposes a separate constraint because each wheel must remain on the supporting steel surface.

Learning-based detectors can identify individual fastener candidates, but they typically require task-specific annotations [28] and do not by themselves recover the group layout or supporting-surface boundary. Depth sensing provides metric local geometry, yet occlusion, oblique viewing, metallic reflections, and missing measurements can fragment both rivet and edge observations. The proposed method, therefore, uses CAD hole layouts to constrain incomplete rivet groups and retains an independent depth branch for member edges (Section 3).

### 2.2. CAD Hole-Layout Template Construction

**Figure 2:**
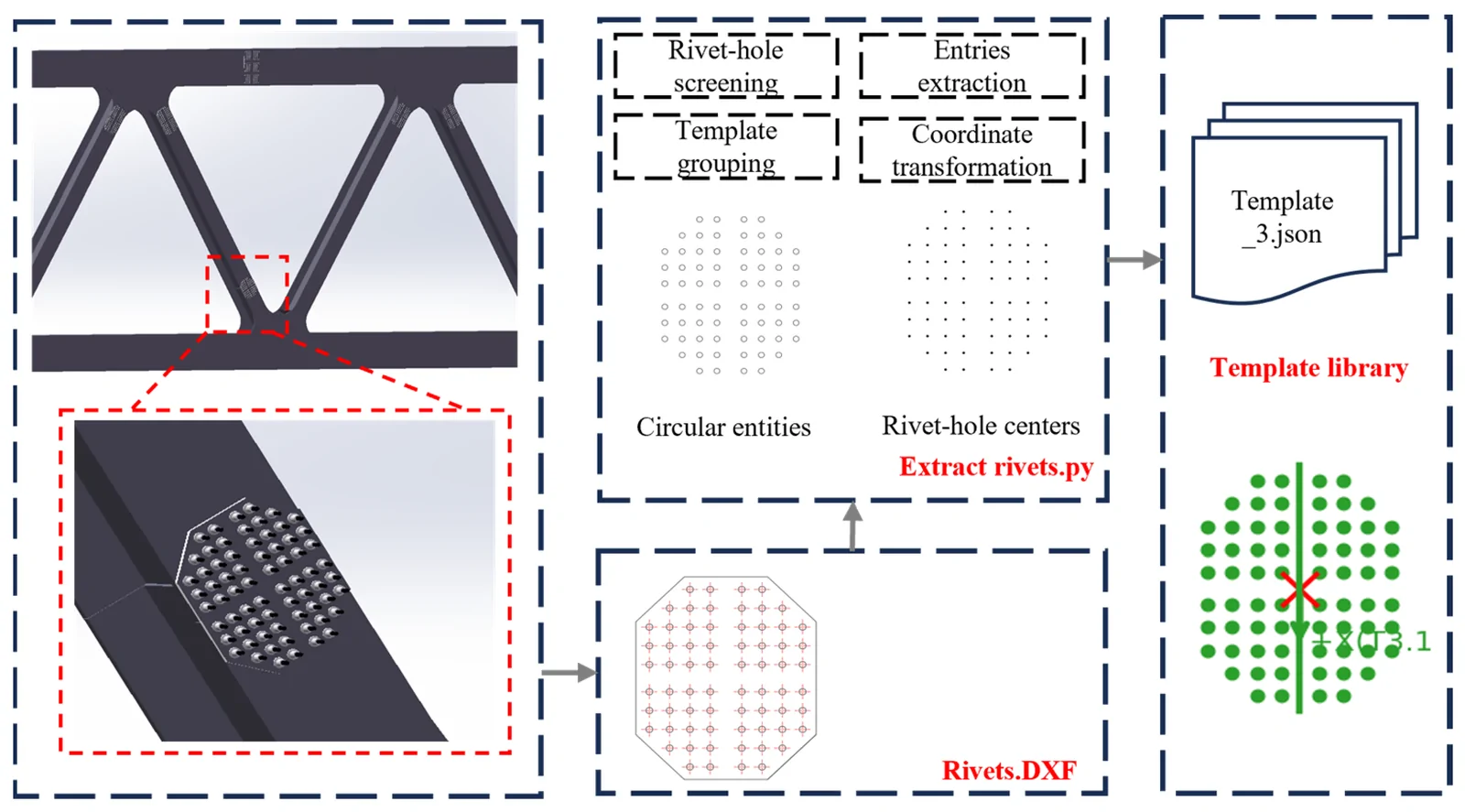
Flowchart of the CAD template extraction procedure.

Bridge CAD drawings in Drawing Exchange Format (DXF) files contain extensive structural information, whereas the online perception module requires only the rivet-hole layouts associated with individual connection details. The offline construction procedure is summarized in Figure 2. Circular entities corresponding to the specified hole diameter are screened from the CAD drawing, and their centers are retained as rivet-hole centers. In parallel, the predefined entry directions associated with these layouts are extracted from the corresponding line entities. The rivet-hole centers are then grouped into individual templates according to their spatial relationships with the entry directions. Finally, each group is transformed from the global CAD coordinate system into a local template coordinate system centered on the hole-layout centroid and aligned with the corresponding entry direction. The resulting local hole-center coordinates and entry-direction information form the template library.

As the robot approaches a rivet group, the forward-facing camera observes the layout progressively from its entry. Each template, therefore, stores one or more candidate entries associated with the widest admissible gaps that satisfy the minimum passage width. These entries define which template rows are expected to enter the observation region first and constrain orientation and correspondence hypotheses during online matching. The row-and-column organization and spacing characteristics derived from the observed rivet candidates are then compared with the stored layouts to retrieve a suitable template for subsequent registration.

## 3. Local Geometric Perception

**Figure 3:**
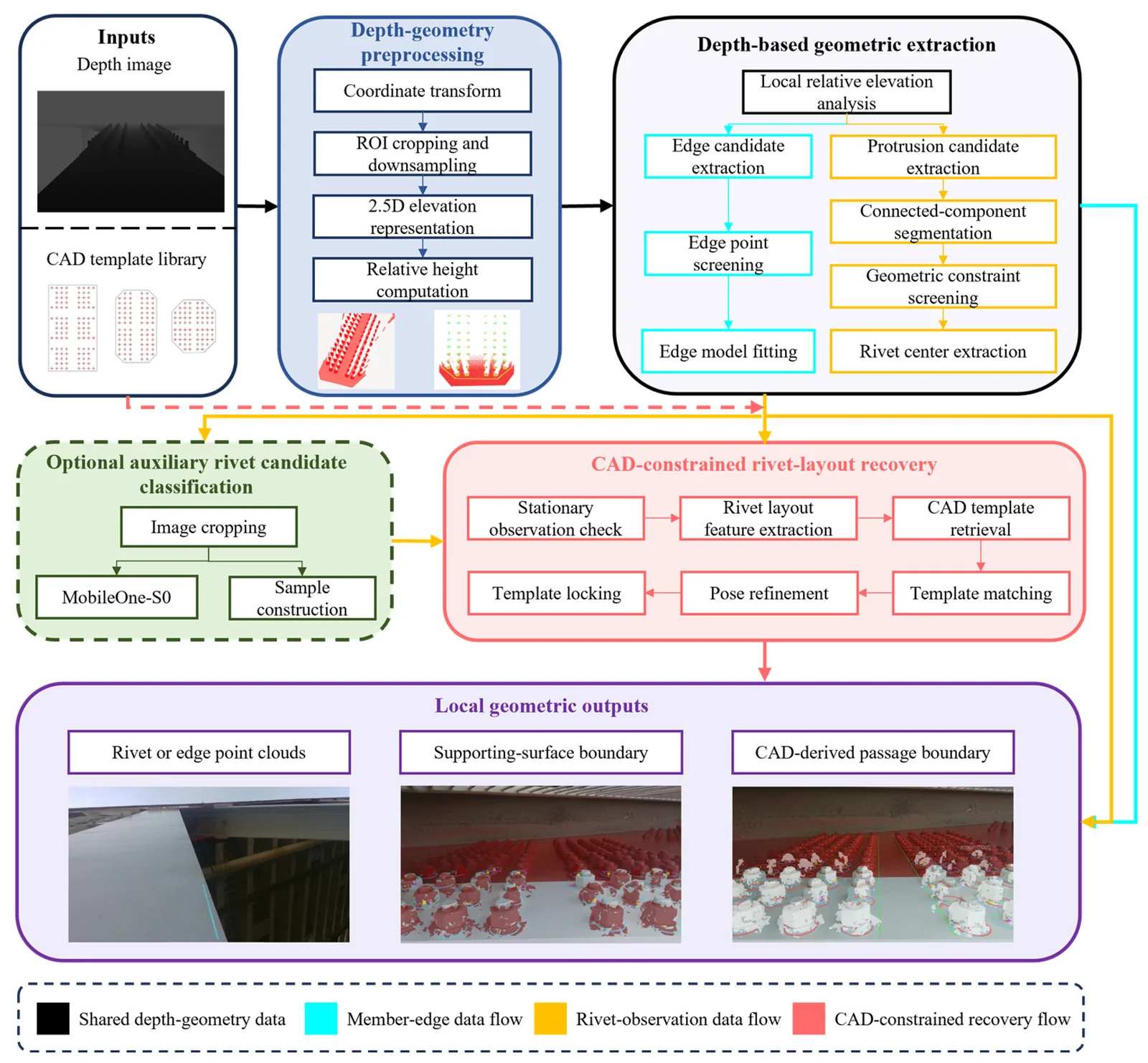
Workflow of the proposed method for local geometric perception.

The method converts incomplete depth observations into two complementary local geometric constraints in the robot body frame (Figure 3, Algorithm 1). The depth-geometry preprocessing stage transforms valid depth measurements into the robot body frame, retains the region of interest (ROI), and organizes the resulting points as a multilayer 2.5D elevation representation. Its relative-height and valid-observation layers provide the common geometric input to both perception branches. The rivet branch extracts protrusion-connected components, screens them using geometric criteria and optional MobileOne-S0 classification, and estimates the centers of the retained rivet candidates. When the robot is stationary and sufficient observations exist, the method uses the partial row-and-column structure to retrieve compatible CAD templates. Planar registration then refines the center-to-hole correspondences and template pose, and consistency verification across stationary frames determines template locking. The locked registration projects the CAD hole layout into the robot body frame, from which the method constructs the theoretical fastener contours and inward-offset passage boundary. The member-edge branch uses the valid supporting region and relative-height transitions to extract boundary candidates and fits a straight-line or transition-curve model to estimate the supporting-surface boundary. The branches share depth preprocessing, but only the rivet branch uses the CAD prior. They produce independent CAD-derived passage and depth-derived supporting-surface boundaries; this study evaluates the two outputs separately and does not fuse them into a navigation map.
**Algorithm 1:** Online local geometric perception pipeline**Input:** depth frame, camera intrinsics, camera-to-body transformation, robot motion state, and CAD template library**Output:** available depth-derived supporting-surface boundary and CAD-derived rivet-passage boundary1:**for** each depth frame **do**2:      Transform valid depth points to the robot body frame, retain the ROI, and construct the multilayer 2.5D representation;
      **Rivet branch**
3:      Extract and geometrically screen protrusion connected components, apply MobileOne-S0 when enabled, and estimate the retained rivet centers;4:      **if** no CAD template is locked and the robot is stationary with sufficient observations **then**5:          Rank CAD templates from partial row-and-column features and select the best candidate;6:          Register the selected candidate and refine its center-to-hole correspondences and pose;7:          **if** the refined hypothesis meets the acceptance criteria for residual, correspondence support, and pose quality **then**8:              Initialize or update the candidate template and pose with the refined hypothesis, and lock both only after three consecutive qualifying estimates;9:          **else**
10:              Reset the candidate state and remain in the search state;11:          **end if**
12:      **end if**
13:          **if** a CAD template is locked **then**14:              Project the locked layout and construct the theoretical rivet contours;15:              Apply the inward offset to the widest inter-column gap and output the candidate passage boundary;16:          **end if**

      **Member-edge branch**
17:      Extract member-edge candidates and fit the supporting-surface boundary when sufficient support is available;18:**end for**

### 3.1. Multilayer 2.5D Elevation Representation

The fixed camera-to-body transformation was determined after installation from mechanical measurements of the translational offsets along the robot body-frame axes and the camera mounting angles relative to these axes. These measurements defined a fixed transformation matrix, which was applied to each raw depth cloud to express the valid points in the robot body frame. The same transformation was used throughout all experiments. Following robot-centric elevation mapping [29,30], the method downsamples the transformed cloud, retains the points within the ROI, and projects them onto an X–Y grid. Multiple elevation statistics are retained in each cell to form the multilayer 2.5D representation. Let $z_{i,j}$ denote the elevations assigned to cell $g_{i,j}$; the layer associated with quantile φ is defined as:

(1) $$E_{i,j}\left(\varphi \right)=Q_{\varphi }(z_{i,j})$$

where $Q_{\varphi }(\cdot )$ denotes the quantile operator. The median layer supports local background estimation; the high and low layers jointly describe protrusion and local variation; and the minimum layer preserves downward responses near an edge. Valid-point counts and a binary mask record observation support, while empty cells remain undefined.

### 3.2. Geometric Feature Extraction from Depth Data

#### 3.2.1. Rivet Candidate Extraction and Center Estimation

Rivet candidates are identified from elevation relative to the immediate supporting surface rather than from absolute elevation. This distinction is necessary because truss members and gusset plates may occupy different elevation levels, although both are locally planar. For each cell $g_{i,j}$, the local background elevation $E_{i,j}^{\mathrm{bg}}$ is estimated as the median of the median elevation layer within a surrounding neighborhood. The protrusion response is defined as:

(2) $$\Delta E_{i,j}=E_{i,j}\left(\varphi_{h}\right)-E_{i,j}^{\mathrm{bg}}$$

Here, $\varphi_{h}$ denotes the high quantile used to construct the protrusion layer.

Cells above the protrusion threshold form a binary mask and are grouped by connected-component labeling [31,32]. Each component is characterized by area, extent, aspect ratio, and mean relative elevation. These criteria jointly suppress small noise clusters, large responses at gusset-plate boundaries, elongated discontinuities, and weak protrusions.

A component centroid can be biased when only part of a rivet is observed. Each retained component is expanded to recover nearby contour evidence. Its centroid provides the initial center estimate, while candidate radii within $\left[r_{\min},r_{\max}\right]$ define the corresponding annular support sets. For each candidate radius $r_{s}$, the support set $G_{s}$ contains contour cells within the corresponding annular band. The center is refined from this support by:

(3) $$\left(x_{s}^{*},y_{s}^{*}\right)=\underset{\left(x,y\right)}{\mathrm{argmin}}\sum_{\left(u,v\right)\in G_{s}}\rho \left(\gamma \sqrt{{\left(u-x\right)}^{2}+{\left(v-y\right)}^{2}}-r_{s}\right)$$

where $\left(u,v\right)$ denotes the grid coordinates of a contour cell, $\left(x,y\right)$ denotes the center estimate in grid coordinates, γ converts grid-index distance to metric distance, and $\rho \left(\cdot \right)$ is the Huber loss:

(4) $$\rho \left(x\right)=\left\{\begin{array}{cc} \frac{1}{2}x^{2}, & \left|x\right|\leq \delta_{\rho }\\ \delta_{\rho }\left(\left|x\right|-\frac{1}{2}\delta_{\rho }\right), & \left|x\right|>\delta_{\rho } \end{array}\right.$$

where $\delta_{\rho }$ denotes the Huber transition threshold. For each candidate radius, iteratively reweighted least squares (IRLS) refines the center by minimizing the objective. The fit score combines contour support and radial residual consistency, and the highest-scoring center-radius pair provides the rivet-center observation for subsequent CAD matching.

#### 3.2.2. Member-Edge Extraction and Model Fitting

Candidate member-edge cells are extracted by jointly evaluating observation availability and relative elevation within a local neighborhood. A valid cell is retained when its elevation is consistent with the supporting surface and the surrounding neighborhood exhibits both a low valid observation ratio and a negative elevation response. These conditions are expressed as:

(5) $${ℳ}_{i,j}^{\mathrm{edge}}=\left\{\begin{array}{ll} 1, & {ℳ}_{i,j}^{\mathrm{depth}}=1,\eta_{i,j}^{\mathrm{obs}}<\tau_{\mathrm{depth}},\Delta E_{i,j}^{\min}<\tau_{\mathrm{neg}},\left|\Delta E_{i,j}\right|<\tau_{\mathrm{plane}}\\ 0, & otherwise \end{array}\right.$$

where ${ℳ}_{i,j}^{\mathrm{edge}}$ and ${ℳ}_{i,j}^{\mathrm{depth}}$ denote the edge-candidate and valid-depth masks, respectively. The quantity $\eta_{i,j}^{\mathrm{obs}}$ gives the valid-cell proportion within the neighborhood. The quantity $\Delta E_{i,j}^{\min}=E_{i,j}^{\min}-E_{i,j}^{\mathrm{bg}}$ is the minimum relative elevation response. The thresholds $\tau_{\mathrm{depth}}$, $\tau_{\mathrm{neg}}$, and $\tau_{\mathrm{plane}}$ constrain observation support, negative elevation, and deviation from the local surface. Small isolated components are removed before the remaining cells are converted into a 3D edge point cloud.

The edge points are ordered along X and fitted using random sample consensus (RANSAC). A straight line represents the boundary when the directional change between the front and rear point subsets remains below the prescribed turning-angle threshold; otherwise, a transition curve represents the local transition. The fitted model defines the depth-derived supporting-surface boundary.

### 3.3. Partial Rivet-Group Recovery with CAD Constraints

During motion, a stop command is issued once the depth observations cover approximately three to four rivet rows. Matching begins after the stationary state has been confirmed. Let $o_{i}$ denote an observed center and $h_{k,j}^{\mathrm{tpl}}$ denote the hole center indexed by j in the local coordinate system of template k.

Because matching begins before the full rivet group is visible, the method compares the observed leading rows with the corresponding entry portion of each template instead of using the total hole count of the complete layout. The layout-compatibility score combines observed counts with spatial features, including adjacent column-center spacings, typical longitudinal and lateral hole spacings, and the maximum lateral gap. The highest-scoring template hypothesis initializes planar Kabsch registration [33] from the corresponding structural centers. A recent valid pose of the same template may be retained to support temporal continuity.

Center-level refinement then evaluates how well each pose hypothesis explains the available rivet evidence. Each observed center is assigned to its nearest transformed hole, pairs beyond the distance threshold are discarded, and only the closest observation is retained for each hole. Let $U^{\left(t\right)}$ denote the resulting unique correspondence set at iteration t. The pose update and root mean square (RMS) residual are:

(6) $$\left(R^{\left(t+1\right)},t^{\left(t+1\right)}\right)=\underset{R\in \mathrm{SO}\left(2\right),t\in {\mathbb{R}}^{2}}{\mathrm{argmin}}{\sum_{\left(i,j\right)\in U^{\left(t\right)}}\left\|o_{i}-\left(Rh_{k,j}^{\mathrm{tpl}}+t\right)\right\|}_{2}^{2}$$

(7) $$e_{\mathrm{rms}}^{\left(t+1\right)}={\left[\frac{1}{N^{u^{\left(t\right)}}}{\sum_{\left(i,j\right)\in U^{\left(t\right)}}\left\|o_{i}-\left(R^{\left(t+1\right)}h_{k,j}^{\mathrm{tpl}}+t^{\left(t+1\right)}\right)\right\|}_{2}^{2}\right]}^{1/2}$$

where $N^{u^{\left(t\right)}}$ denotes the number of unique correspondences. Correspondence assignment and planar registration alternate for a fixed number of refinement iterations and stop early if no valid pose update is available.

After refinement, the selected template is transformed into the robot body frame. Holes within the expanded longitudinal and lateral bounds of the active observations define the current template-support set. Holes outside these bounds are excluded from the coverage calculation and are not counted as missed observations. Only detected centers retained in the correspondence set are treated as observations; the remaining transformed hole centers belong to the template-completed layout.

The final residual measures geometric agreement, but a small correspondence subset can also produce a low value. Each refined pose is, therefore, evaluated by fitting accuracy, correspondence coverage, and support size. Let $N^{u}$ be the final number of unique correspondences, $N^{o}$ the number of active observations, and $N^{\mathrm{vis}}$ the number of visible template holes. The observation explanation ratio and visible-hole support ratio are $P=N^{u}/N^{o}$ and $C=N^{u}/N^{\mathrm{vis}}$, respectively. The pose quality score is:

(8) $$S=\exp\left[-{\left(\frac{e_{\mathrm{rms}}}{\sigma_{e}}\right)}^{2}\right]\cdot \frac{2PC}{P+C}\cdot \left[\alpha +\left(1-\alpha \right)\min\left(1,\frac{N^{u}}{N_{\min}^{u}}\right)\right]$$

where $\sigma_{e}$ controls the decay with the RMS registration residual and $N_{\min}^{u}$ is the correspondence count at which the support factor saturates. α sets the minimum contribution of this factor, and the final score is clipped to $\left[0,1\right]$. Unlike the layout compatibility score used for template retrieval, S evaluates the geometric reliability of a refined pose within the currently observable region. The highest-scoring pose hypothesis proceeds to temporal verification only if it satisfies the minimum correspondence-count and pose-quality criteria. If no hypothesis is acceptable before locking, the system remains in the search state and does not construct a CAD-derived passage boundary.

Template matching relies on partial observations, so the method confirms the template pose across three consecutive qualifying stationary estimates. The first qualifying estimate initializes a candidate pose and sets the count to one. Each of the next two consecutive estimates must select the same template and satisfy the update-quality criteria, including the prescribed translation and rotation bounds relative to the current candidate. For each of these two accepted updates, the method applies a small correction to the candidate pose, which becomes the reference for the next estimate. Upon accepting the third consecutive qualifying estimate, the method locks the candidate as the recovered template pose. A non-qualifying estimate resets the count before the confirmation process resumes.

Once stationary convergence is reached, the local reference frame L is defined as a fixed local frame coincident with the robot body frame at lock time. The converged transformation fixes all holes of the selected template in L. These locked holes constitute the template-completed layout. Updating this frame after subsequent robot motion is outside the present evaluation.

### 3.4. Rivet-Passage Boundary Construction

The locked layout contains discrete hole centers and must be converted into a representation of rivet occupancy. A circular theoretical contour with the maximum rivet outer radius $r_{\max}$ is projected around each locked hole center. Among adjacent rivet columns, the widest gap satisfying the minimum passage width is selected. Let j* and j*+1 denote the two ordered columns that bound this gap. Their lateral limits in L are:

(9) $$\left\{\begin{array}{c} y_{\mathrm{lower}}^{L}=y_{j*}^{L}+r_{\max}+\Delta_{\mathrm{shrink}}\\ y_{\mathrm{upper}}^{L}=y_{j*+1}^{L}-r_{\max}-\Delta_{\mathrm{shrink}} \end{array}\right.$$

where $\Delta_{\mathrm{shrink}}$ is a fixed inward boundary offset. It narrows the nominal passage to reduce the influence of residual template registration error, fastener dimensional variation, and depth measurement uncertainty.

The minimum and maximum X coordinates of the template-completed layout define the longitudinal range after expansion by the rivet radius and a prescribed two-sided extension. This extension carries the boundary beyond both terminal rows; it is not an oriented forward-motion hypothesis. Together, the lateral and longitudinal limits define the CAD-derived local rivet-passage region for the selected template and inward offset in the lock-time local frame.

### 3.5. CAD-Assisted Annotation and Optional Candidate Screening

After template locking, the registered hole layout supplies a structural prior aligned with the corresponding depth observation. Following the general deterministic auto-labeling principle of [34], this prior is combined with local depth geometry to generate pseudo-labeled samples with limited manual intervention.

**Figure 4:**
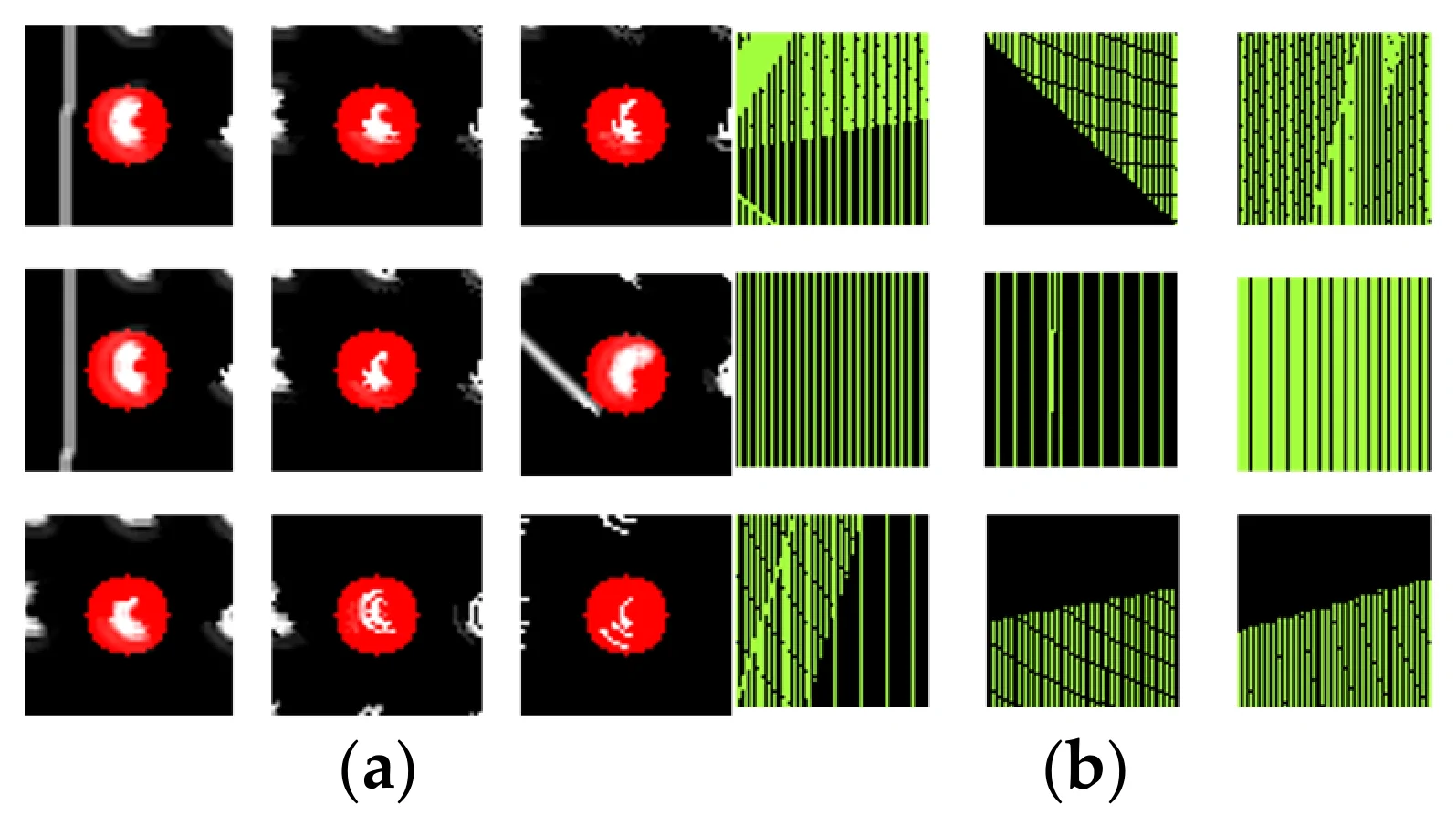
Examples of training samples: (**a**) positive samples and (**b**) negative samples.

Training samples are collected from stationary frames after CAD convergence. Each candidate is represented by a multichannel patch from the 2.5D representation, comprising relative elevation, observation validity, and local geometric variation. A candidate response coincident with a visible hole in the registered template forms a positive sample. A response without template support receives a negative label only when it lies in a reliably observed region and remains inconsistent under stable locking; patches from locally flat surfaces provide additional negative samples. Thus, the CAD prior constrains the interpretation of depth evidence rather than directly replacing that evidence. Representative samples are shown in Figure 4.

The pseudo-labeled samples can train a lightweight classifier based on the MobileOne-S0 backbone [35]. During subsequent inference, the optional classifier assigns a rivet confidence to each geometrically extracted candidate and may suppress some spurious responses caused by metallic reflections, depth noise, or unrelated surface discontinuities. Because the present study does not report a held-out quantitative assessment, this module is excluded from the primary performance claims.

## 4. Experiments

### 4.1. Experimental Platforms

**Figure 5:**
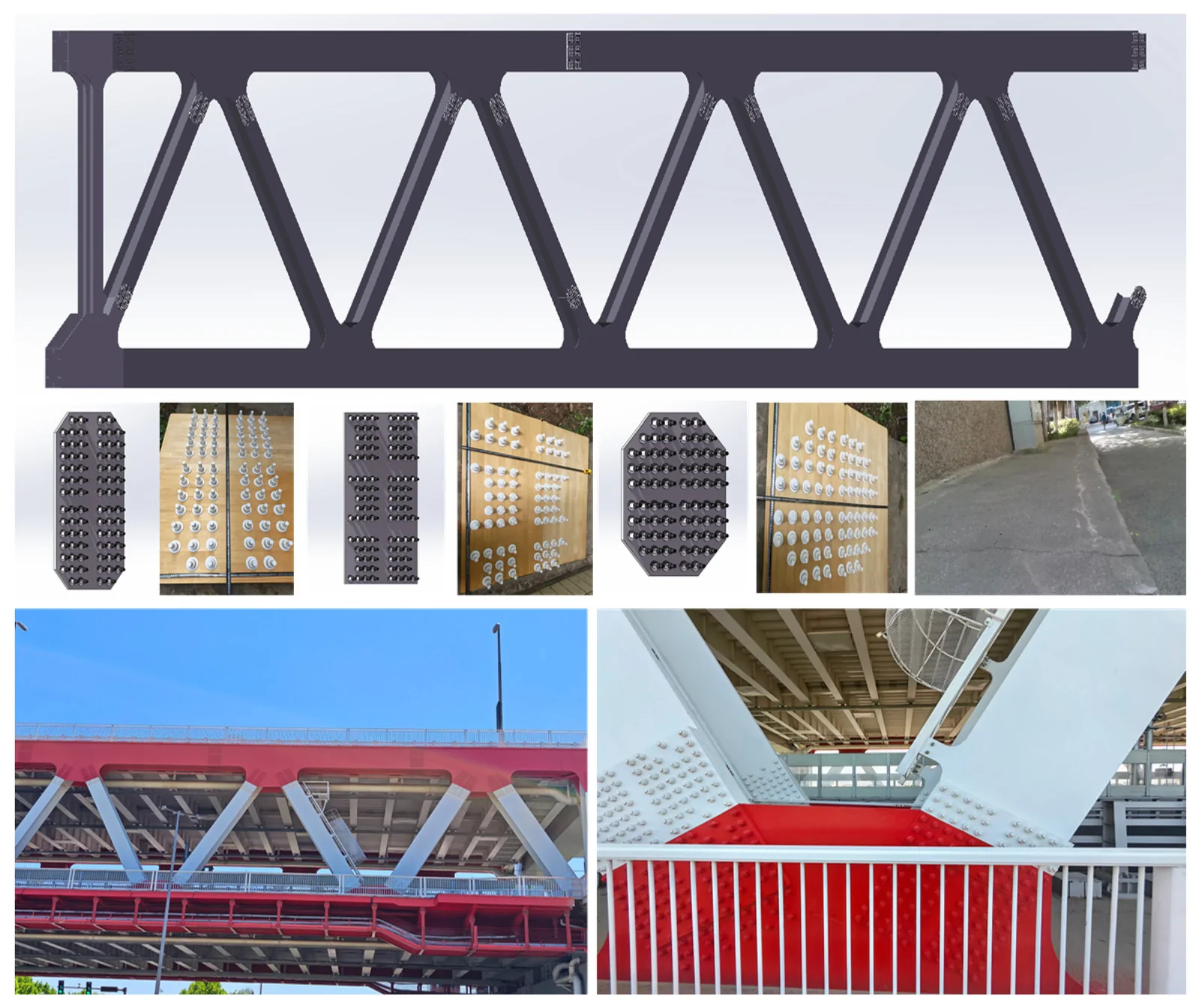
Experimental platforms: simulated double-deck steel truss bridge (**top**); simulation and laboratory mock-ups of the three rivet-group templates and the member-edge mock-up (**middle**); field bridge overview (**bottom left**) and local details (**bottom right**).

The evaluation combined simulation, laboratory mock-ups, and one field case (Figure 5). The Gazebo simulation covered three typical rivet-hole layout types from the target bridge drawings, together with straight and transition member edges. The known model coordinates and Gazebo-reported robot pose established the ground-truth rivet centers and member-edge geometry in the robot body frame. These references supported the quantitative evaluation of center localization, passage-boundary clearance, and member-edge fitting. The laboratory mock-ups reproduced the three rivet layouts with 3D-printed rivets and used a stepped platform to represent a straight member edge, providing controlled conditions for testing template locking, passage-boundary generation, and member-edge fitting. The field experiment examined these functions using one bolted connection and one straight member edge on another double-deck steel truss bridge. Both physical platforms lacked independent millimeter-level ground truth. The laboratory results, therefore, provide controlled functional evidence and qualitative overlays, while the field results provide single-case feasibility evidence from real bridge depth observations.

**Figure 6:**
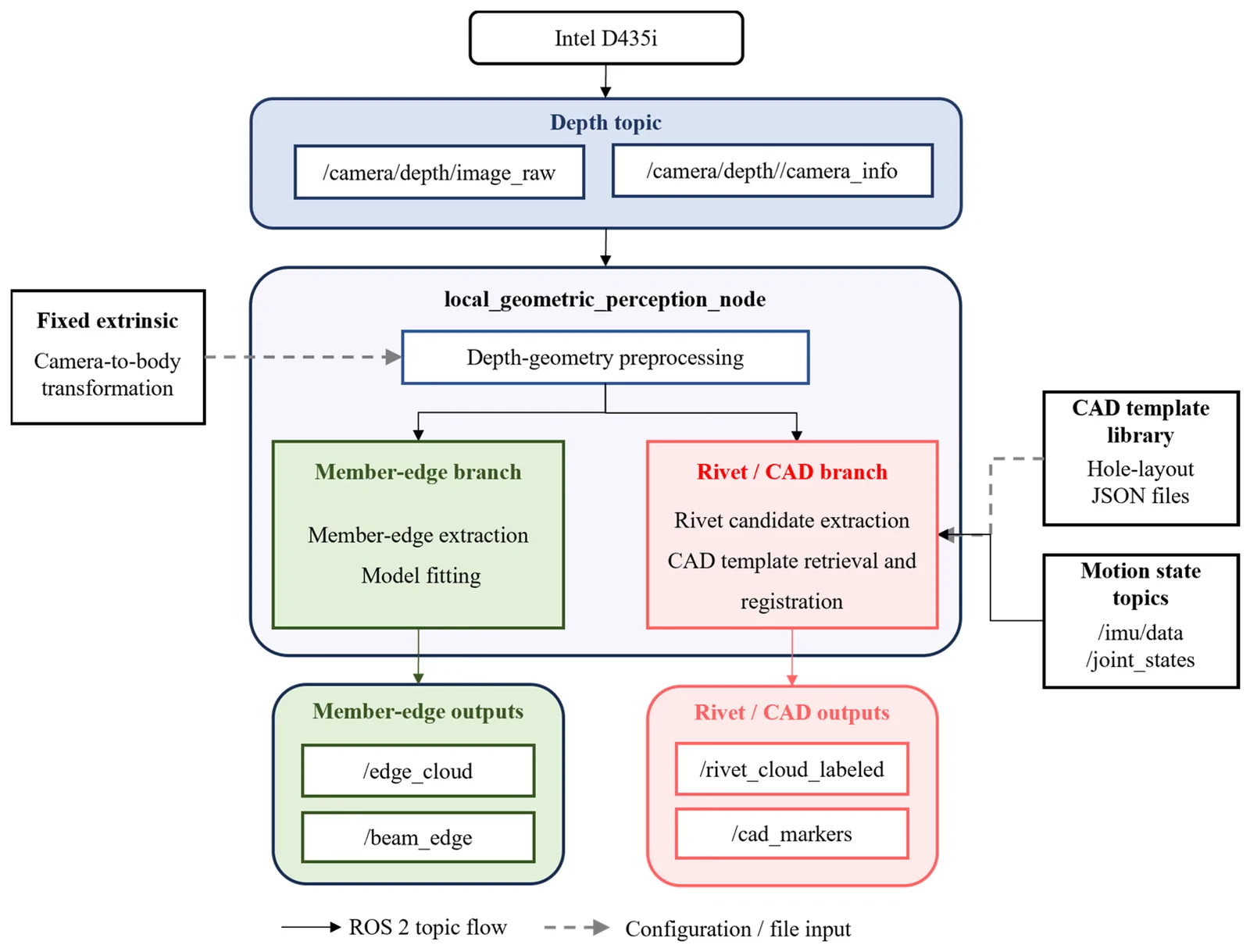
ROS 2 architecture and data flow.

The system issued the stop command automatically in simulation, while the operator triggered it manually in the laboratory and field experiments. The algorithm ran in a VMware virtual machine on an AMD Ryzen 9 7945HX laptop. The virtual machine used 16 virtual cores and 32 GB of RAM and ran Ubuntu 22.04 and Robot Operating System 2 (ROS 2) Humble without GPU passthrough. Figure 6 presents the ROS 2 node structure. Table 1 summarizes the geometric settings used in the experiments. Table A1 lists the fixed algorithm settings, and Table A2 reports the depth-camera specifications and acquisition settings used in the physical experiments. The values for field of view, operating range, and nominal depth accuracy in Table A2 come from the manufacturer’s specifications. Depth measurement uncertainty was not independently quantified under the physical test conditions. The geometric and algorithm settings were selected based on the target geometry, depth-sensing characteristics, experimental conditions, and engineering considerations.

### 4.2. CAD-Constrained Rivet-Group Recovery

#### 4.2.1. Simulation Experiment

**Figure 7:**
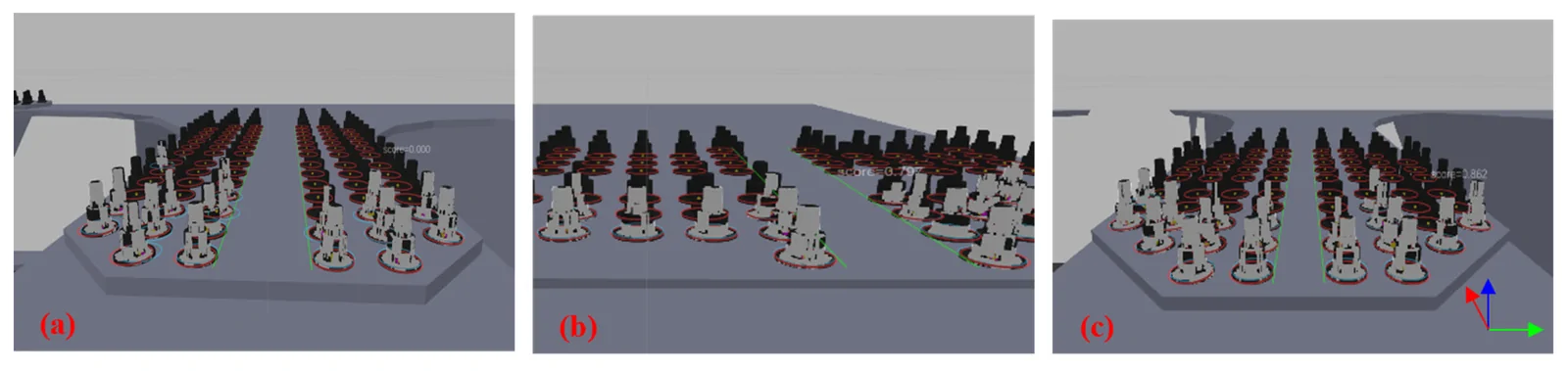
Simulation results for three templates: (**a**) Template 1; (**b**) Template 2; and (**c**) Template 3. White points show the retained rivet point cloud, blue circles show contours from geometric extraction, red circles show theoretical contours projected from the locked CAD layout, and green lines show the rivet-passage boundaries.

For each rivet template, 15 trials were conducted with different stopping positions and viewing orientations to vary the visible rivet distribution and partial-view geometry. The deviation from the nominal passage axis was kept within ±15°. The repeated trials were used to assess the consistency of template recovery under these observation variations. With the correct layout present in the three-template library, the method selected and locked the correct template and generated the corresponding passage boundary in all 45 trials, corresponding to 45/45 correct selections, 45/45 successful locks, and 45/45 successful boundary outputs. Representative outputs are shown in Figure 7.

**Figure 8:**
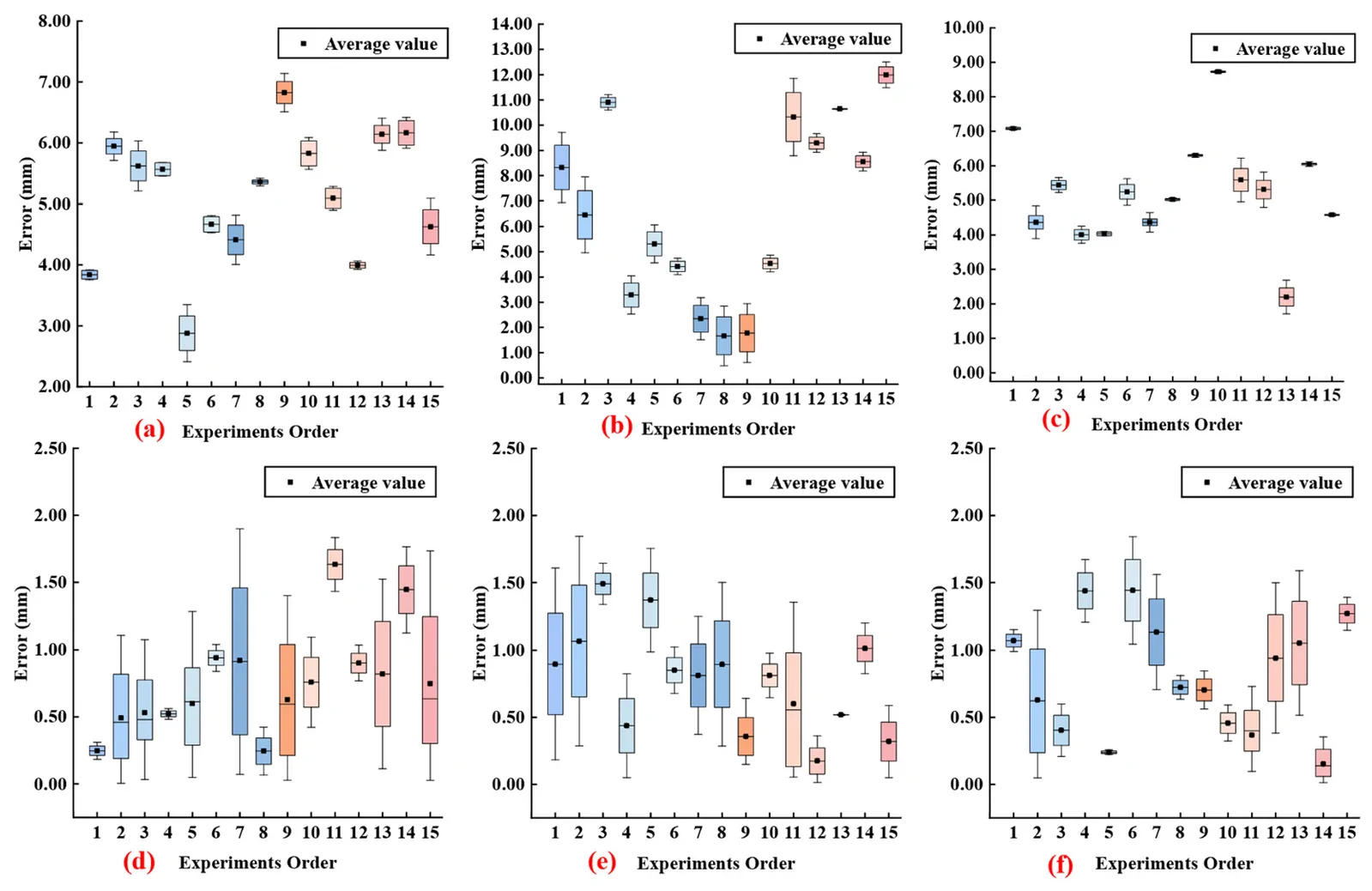
Error distributions for the three templates: (**a**–**c**) errors in the X direction and (**d**–**f**) errors in the Y direction.

Localization was evaluated separately along the robot travel direction (X) and lateral direction (Y). For each trial, mean absolute error (MAE) and root mean square error (RMSE) were calculated between the transformed CAD hole centers and the ground-truth rivet centers. Boundary clearance was defined as the minimum distance from a generated passage boundary to a ground-truth rivet center minus the corresponding theoretical radius; a positive value means that the boundary remains outside that theoretical contour. Figure 8 shows the trial distributions, and Table 2 reports values averaged over 15 trials.

Under the exact-template simulation condition, mean Y-direction MAE and RMSE remained below 1 mm for all three templates. X-direction MAE ranged from 5.130 to 6.655 mm, and X-direction RMSE ranged from 5.135 to 6.697 mm. Minimum clearance was positive in every trial; the mean minimum clearance across templates ranged from 0.792 to 0.921 mm. Mean locking time ranged from 4.58 to 10.81 s.

**Figure 9:**
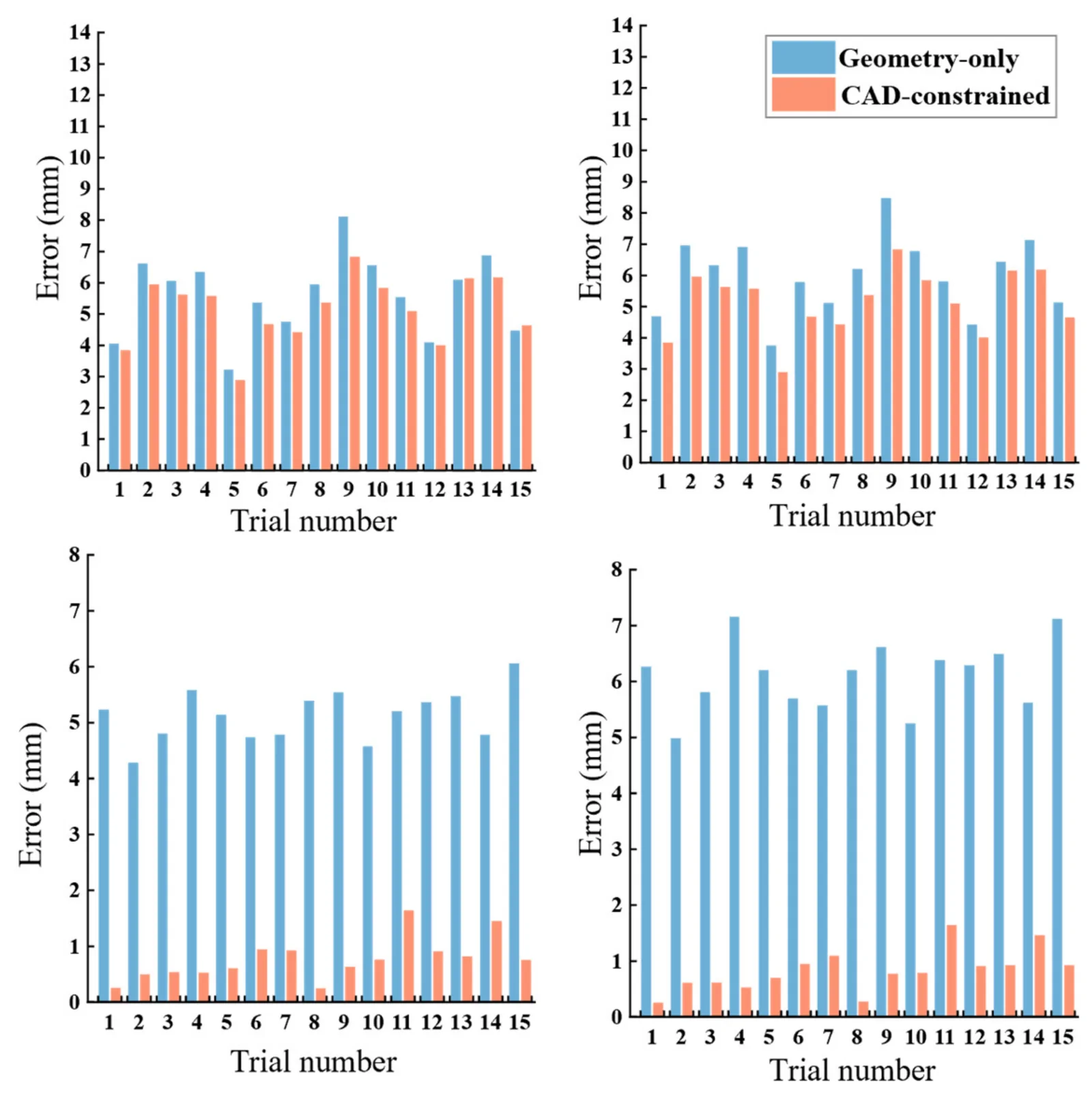
Comparison of localization errors for Template 1. The top row shows MAE-X and RMSE-X, and the bottom row shows MAE-Y and RMSE-Y.

**Figure 10:**
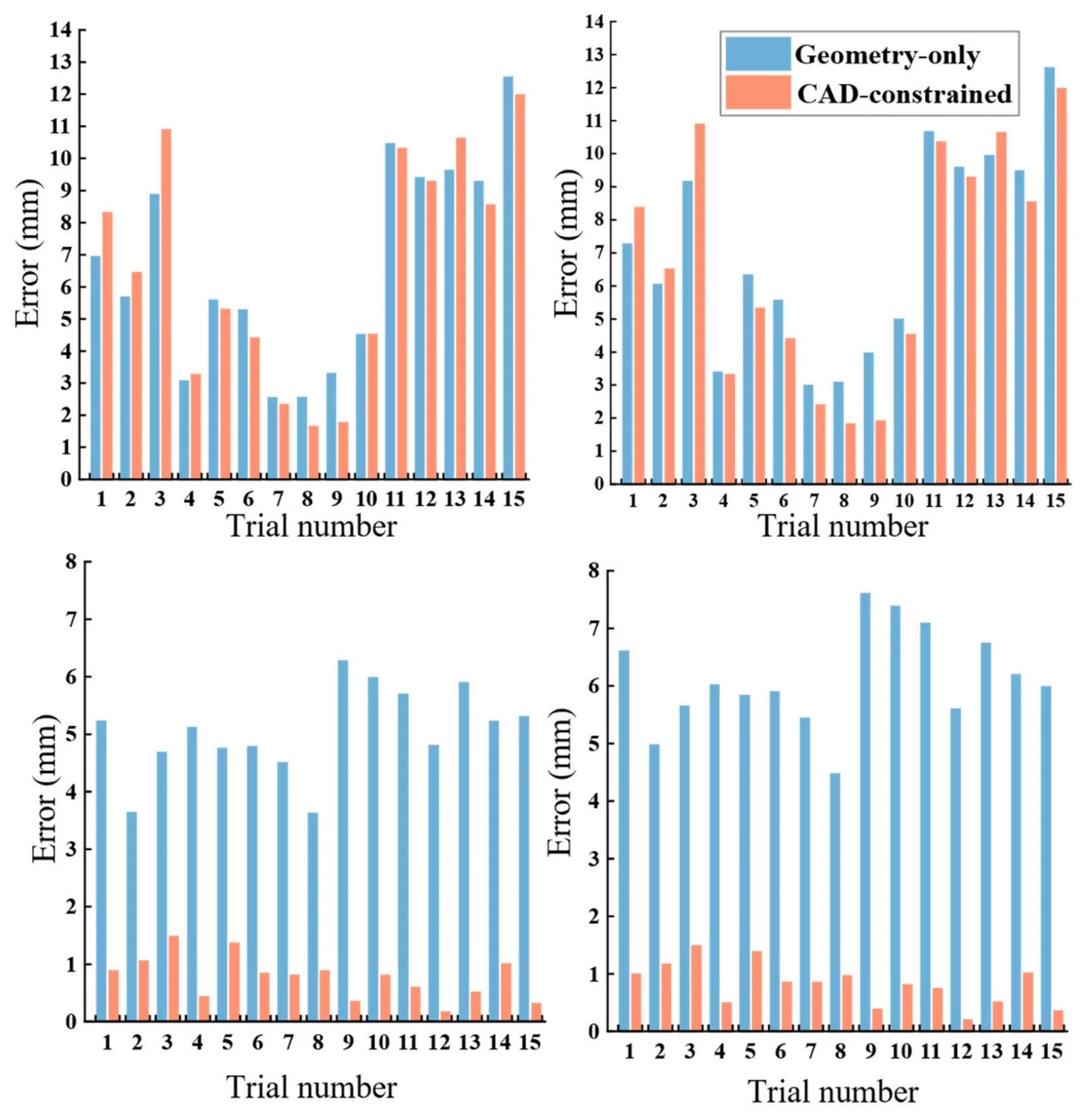
Comparison of localization errors for Template 2. The top row shows MAE-X and RMSE-X, and the bottom row shows MAE-Y and RMSE-Y.

**Figure 11:**
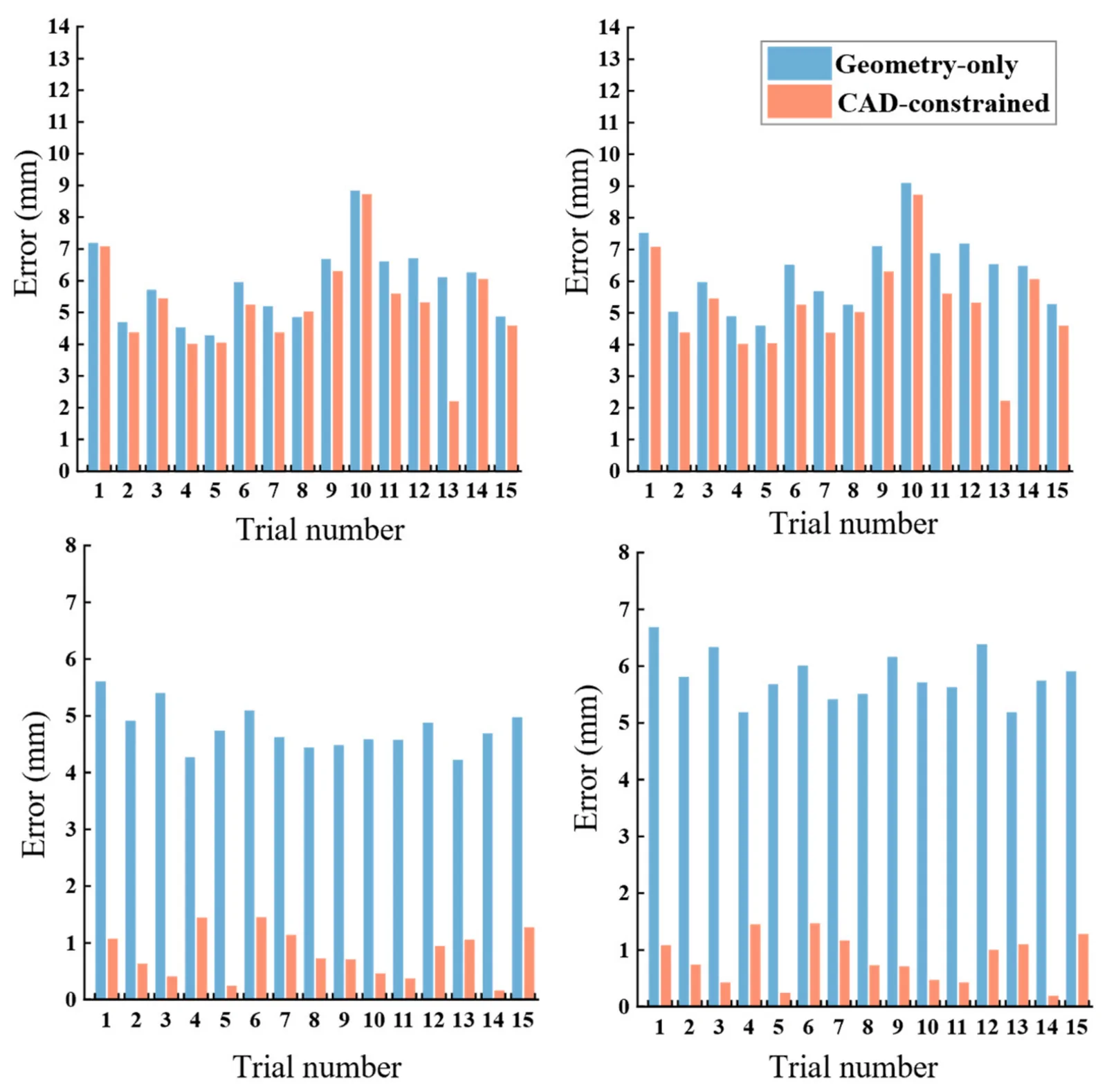
Comparison of localization errors for Template 3. The top row shows MAE-X and RMSE-X, and the bottom row shows MAE-Y and RMSE-Y.

Alternative geometric front ends would change candidate extraction, while methods that estimate only individual fastener centers or contours do not recover the group-level geometry required for passage-boundary construction. To isolate the contribution of the CAD constraint, a geometry-only ablation baseline was constructed from centers obtained directly via geometric extraction and circle fitting and compared with the locked CAD hole centers. Both methods were evaluated on the same depth observations and the same visible rivets with ground-truth correspondences. Table 3 reports MAE and RMSE along X and Y, averaged over 15 trials for each template. Relative to the geometry-only ablation baseline, CAD-constrained recovery reduced lateral MAE by 83.17–85.14% and lateral RMSE by 85.79–86.55%. The corresponding reductions along X were 0.01–11.38% for MAE and 4.56–16.54% for RMSE. Figure 9, Figure 10 and Figure 11 show the trial-level comparisons.

#### 4.2.2. Laboratory and Single-Case Field Feasibility Evaluation

**Figure 12:**
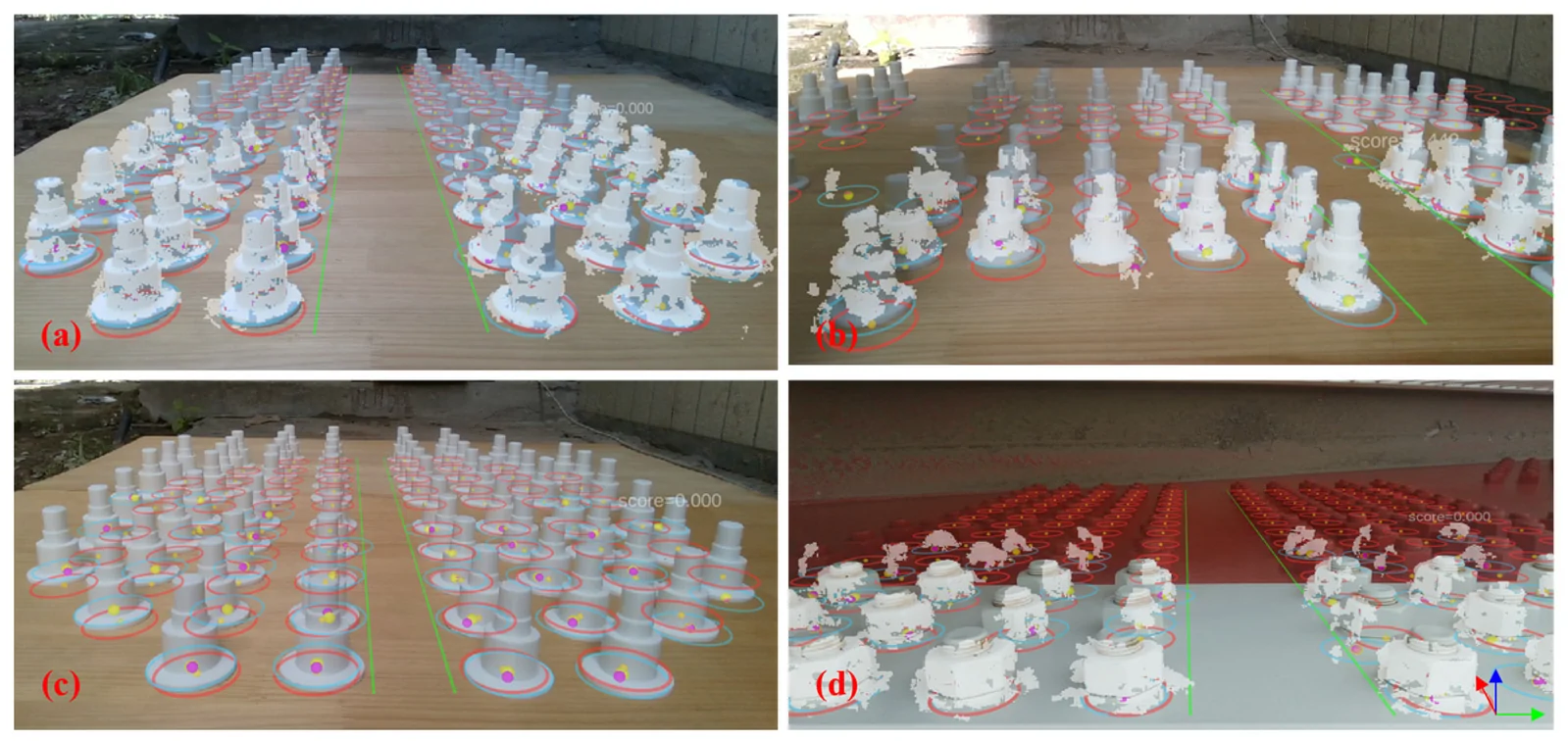
Representative laboratory results for Templates 1–3 ((**a**,**b**) and (**c**), respectively) and the single-case field bolt-group result (**d**).

Ten repeated trials were performed for each laboratory template. In the laboratory, the workflow selected the correct template in every trial; qualitative inspection of the overlays found no visible boundary intrusion. Mean locking times were 9.24, 28.51, and 12.90 s for Templates 1–3, respectively. These observations show functional template locking and boundary generation for the tested mock-ups; they do not provide millimeter-level physical accuracy. The field evaluation comprised ten repeated observations of one bolted connection configuration. A case-specific layout prior was constructed from manual measurements of the same bolt group. Although the field fasteners differed from the rivets in local shape and protrusion geometry, both were represented by hole-center layouts during downstream CAD matching. Accordingly, CAD-based matching, locking, and passage-boundary construction remained unchanged, while only fastener-specific extraction parameters were adjusted for the bolt geometry. In all ten observations, the workflow matched this case-specific prior and generated a template-completed bolt layout; qualitative overlay inspection found no visible boundary intrusion. Figure 12 shows representative results.

For laboratory Template 1, a single-factor test varied the inward offset to examine its effect on the reported passage width and boundary overlap. A trial met the non-intrusion criterion when no part of the generated boundary visibly overlapped an observed rivet region. The number of non-intrusive trials increased from 7/10 at 4 and 6 mm to 9/10 at 8 and 10 mm and 10/10 at 12 mm (Table 4). Accordingly, the laboratory and field evaluations used a 12 mm inward offset.

**Figure 13:**
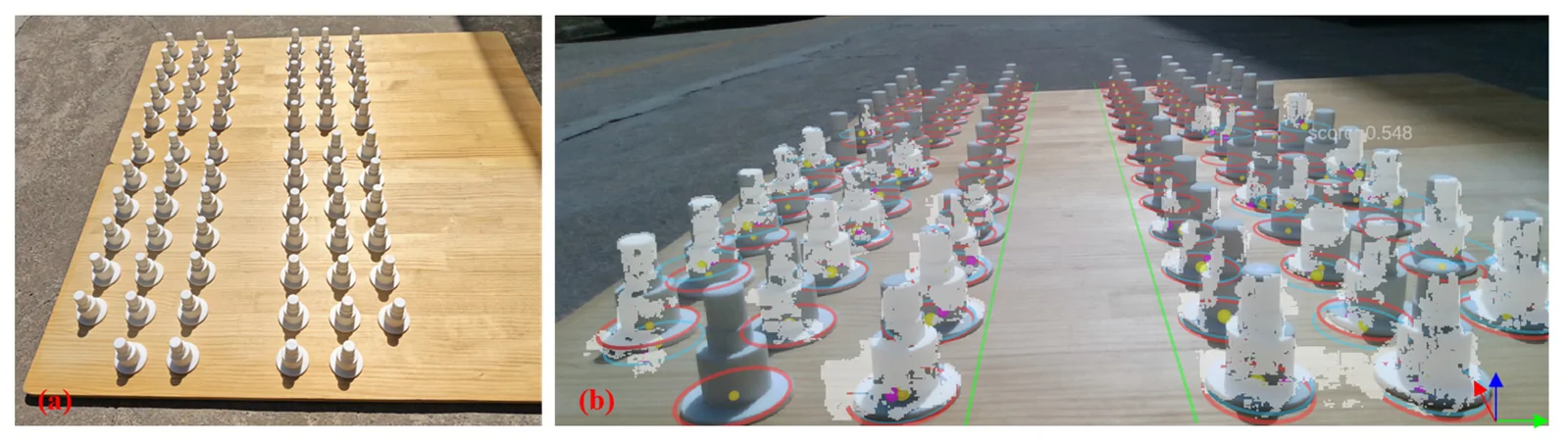
Direct-sunlight laboratory test for Template 1: (**a**) experimental scene and (**b**) representative perception result.

The method uses D435i depth measurements rather than RGB intensity, so visible-light shadows do not directly affect the geometric pipeline. Strong sunlight can still disturb the infrared stereo images and cause noisy or missing depth measurements, especially on reflective surfaces. To examine this condition, the laboratory evaluation included ten additional direct-sunlight trials using Template 1. The method locked the correct template and generated a passage boundary in all ten trials, and every boundary met the same qualitative non-intrusion criterion. Although the depth observations showed greater local disturbance than under normal illumination, they retained sufficient geometric evidence for template locking and boundary construction (Figure 13). These results show functional feasibility under the tested direct-sunlight condition.

**Figure 14:**
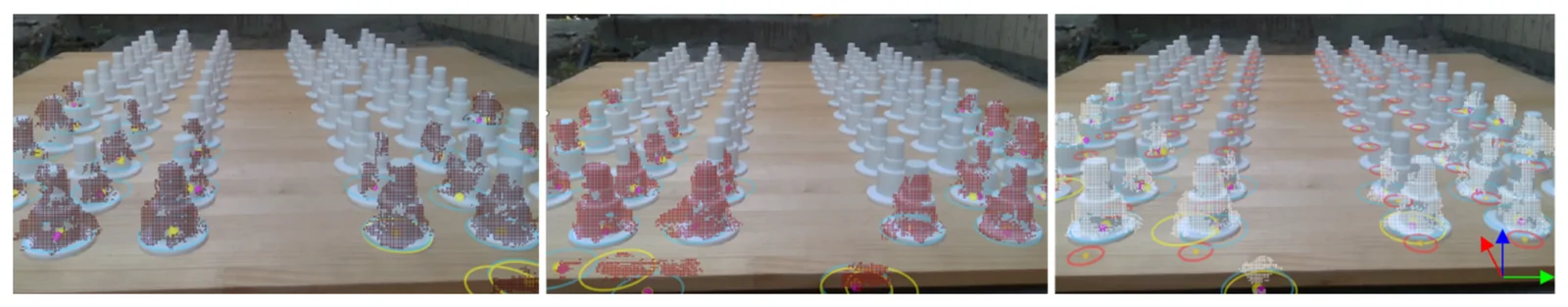
Candidate-level auxiliary classification results. The yellow circles indicate candidate regions classified as non-rivets and removed by the classification network.

The optional classifier was examined only as an exploratory candidate-screening module. The automatically annotated dataset contained 2179 depth patches collected after stable CAD locking in simulation and laboratory experiments: 1758 positive and 421 negative samples. The classifier suppressed some spurious geometric responses but also rejected some valid rivet candidates. Because no held-out quantitative evaluation was conducted, the classifier is not treated as a validated component of the core perception method. Figure 14 shows qualitative examples.

### 4.3. Member-Edge Perception

Member-edge perception was first evaluated against simulation ground truth for straight and transition geometries, with three test groups for each geometry. Data were sampled at 0.5 s intervals. A fit was considered valid when the extracted edge points met the minimum support requirement and the fitted straight-line or transition-curve model satisfied the prescribed criteria. For each valid simulation frame, the geometric distances between the fitted boundary and the ground-truth member edge were calculated, and their MAE and RMSE were used as the boundary-error metrics.

All sampled simulation frames produced valid fits. The straight-edge groups yielded an MAE of 21.380 mm and an RMSE of 22.262 mm, whereas the transition-edge groups yielded an MAE of 30.487 mm and an RMSE of 35.635 mm (Table 5).

**Figure 15:**
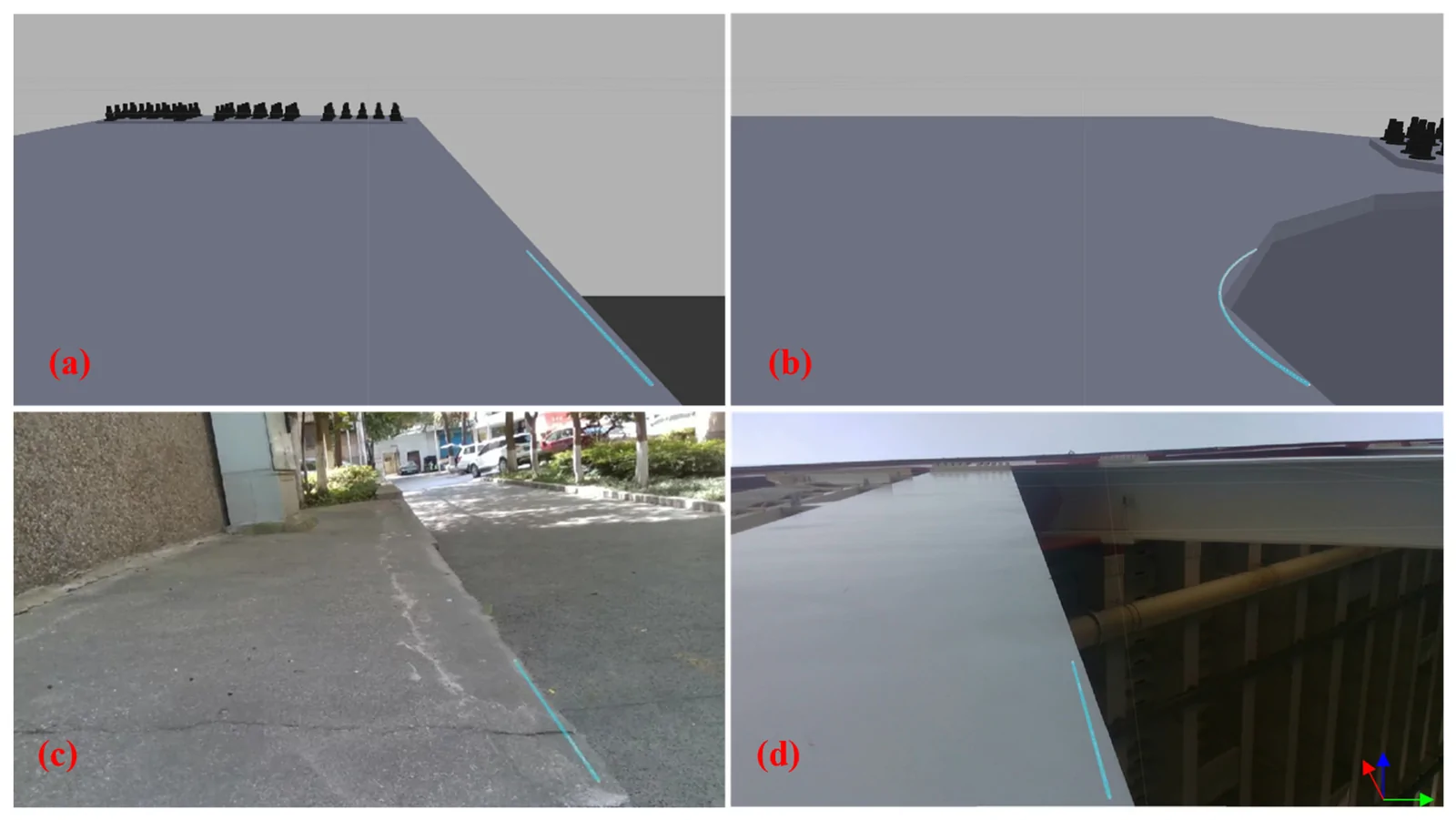
Member-edge perception across experimental settings: simulated straight and transition edges (**a**,**b**), laboratory straight edge (**c**), and field straight edge (**d**). White points show the extracted edge cloud, and blue or cyan lines show the fitted supporting-surface boundary.

Physical testing considered straight edges because the transition geometry could not be reproduced reliably in the laboratory mock-up. Valid fits were obtained in 1124 of 1325 laboratory frames (84.83%) and 928 of 1166 field frames (79.59%). A frame was considered invalid when it contained insufficient accepted edge points or did not satisfy the prescribed model-fitting criteria. Because the physical platforms lacked independently measured three-dimensional edge ground truth, the laboratory and field evaluations report valid-fit rates and qualitative overlays rather than millimeter-level edge errors. Across the simulation and physical tests, mean processing time for the member-edge branch ranged from 20.36 to 23.37 ms. Figure 15 shows representative outputs.

## 5. Discussion

The proposed local geometric perception method addresses two geometric constraints encountered by climbing robots on double-deck steel truss bridges. The CAD branch constructs a rivet-passage boundary from the locked CAD layout, whereas the depth branch estimates the termination of the supporting surface near a member edge. Both outputs provide local geometric inputs for subsequent motion planning.

Under exact-template simulation conditions, the CAD-constrained branch produced its clearest benefit in lateral center estimation. Relative to the geometry-only ablation baseline, lateral MAE decreased by 83.17–85.14%, and lateral RMSE decreased by 85.79–86.55% (Table 3). This directional difference reflects the stronger lateral constraint supplied by fixed inter-hole spacings, whereas partial contours and oblique viewing leave longitudinal estimates more sensitive to missing depth. By retaining the same partial depth observations and geometric front end while omitting the CAD layout constraint, the geometry-only ablation baseline isolates the regularization introduced by the CAD prior. The resulting improvements, therefore, show the value of CAD-constrained recovery within the tested exact-template conditions.

The inward-offset study indicates that an explicit geometric allowance can moderate residual mismatch between the CAD-derived boundary and physical depth observations. The progressive reduction in visible intrusion motivated the 12 mm setting used in the physical evaluation. Its continued non-intrusive behavior under direct sunlight, despite greater disturbance in the depth observations, suggests that the selected offset accommodated the sensing variation encountered in that test. This result supports CAD-derived passage-boundary generation under the tested sunlight condition. The single-template qualitative evaluation does not establish performance across uneven illumination conditions. The 12 mm value remains an empirical parameter for the tested configuration and does not define a validated robot-level safety margin.

Member-edge estimation does not use a CAD layout prior and instead relies on local depth features and geometric model fitting. This dependence on incomplete local observations likely contributes to the error magnitude reported in Table 5, while transition edges are more sensitive to changes in boundary direction. In the representative results shown in Figure 15, the fitted boundaries lie toward the interior of the supporting surface. However, this tendency was observed only in the illustrated cases and does not provide a quantitative criterion for determining whether the errors are acceptable for robot passage. Because the present study did not define a robot-specific navigation clearance threshold or evaluate the boundary estimates in closed-loop traversal, the results in Table 5 characterize boundary-estimation accuracy rather than demonstrating that the achieved accuracy is sufficient for safe operation. The physical valid-fit rates also show that some frames lacked a boundary output. Because the temporal distribution of these frames was not evaluated, continuous operation will require improved fitting and explicit temporal handling.

The physical experiments show that both branches can produce their intended geometric outputs from real depth observations, extending the evidence beyond simulation. The physical platforms lacked independent millimeter-level ground truth, so these tests establish functional repeatability but cannot provide millimeter-level physical error estimates. The laboratory mock-ups isolate repeatability under controlled geometry, and the stepped platform edge reproduces support-surface termination without replicating the full sensing conditions of a steel member. The field test on another double-deck steel truss bridge adds real surface and ambient conditions. The field evaluation repeatedly observed one bolt group and one straight edge using a prior measured from the same connection. The result supports single-case feasibility, while generalization across field configurations remains untested.

The main limitation is the dependence of CAD matching on agreement between the template library and the as-built fastener layout. Construction tolerances, deformation, missing or displaced fasteners, and drawing discrepancies may degrade registration. Expanding the library would improve layout coverage but could increase ambiguity among similar templates under partial visibility. A closely matching unlisted layout could also be falsely accepted because the method lacks an independent open-set rejection stage. No separate target-based extrinsic calibration or repeatability assessment was performed; therefore, the translational and rotational uncertainty of the mechanically determined camera-to-body transformation was not quantified. The sensitivity of the results to the fixed algorithm parameters was also not systematically evaluated. The two geometric constraints were evaluated separately, without integration into closed-loop planning and control or evaluation of magnetic adhesion and traversal safety. Future work should establish physical ground truth, evaluate as-built and unknown-layout cases, quantify calibration and parameter sensitivity, and improve temporal edge tracking before integrating both constraints into a complete robotic system.

## 6. Conclusions

This study developed a local geometric perception method for climbing robots on double-deck steel truss bridges. The method organizes depth observations into a shared multilayer 2.5D representation. The CAD branch recovers a local rivet-group layout from partial center observations and constructs a rivet-passage boundary. The member-edge branch fits the termination of the supporting surface from local depth geometry.

Under exact-template simulation conditions, CAD-constrained recovery reduced lateral MAE by 83.17–85.14% and lateral RMSE by 85.79–86.55% relative to the geometry-only ablation baseline. Mean lateral errors remained below 1 mm, and every simulated boundary retained positive theoretical clearance. Laboratory tests achieved template locking for three layouts, and qualitative inspection of the overlays found no visible boundary intrusion. Ten stationary field checks matched one manually measured bolt-group prior. The field member-edge branch returned valid fits in 928 of 1166 frames (79.59%), with a mean branch processing time of 23.37 ms.

The evidence supports CAD hole-layout templates and depth geometry as complementary sources of local constraints for the evaluated layouts and scenes. Physical geometric accuracy, broader as-built variability, full-pipeline real-time performance, and autonomous traversal were not evaluated. These questions must be resolved before the method can support a claim of navigation safety.

## Figures and Tables

**Table 1:** Geometric settings used in the experiments.

Parameter	Description	Value
[$x_{\min}^{\mathrm{roi}}$,$x_{\max}^{\mathrm{roi}}$]	ROI in X direction	[0.35, 1.50] m
[$y_{\min}^{\mathrm{roi}}$,$y_{\max}^{\mathrm{roi}}$]	ROI in Y direction	[−0.60, 0.60] m
γ	Grid resolution	2.5 mm
[$r_{\min},r_{\max}$]	Rivet outer-contour radius range	[20.0, 31.5] mm
$\Delta_{\mathrm{shrink}}$	Simulation inward boundary offset	4.0 mm
$\Delta_{\mathrm{shrink}}$	Laboratory inward boundary offset	12.0 mm
$\Delta_{\mathrm{shrink}}$	Field inward boundary offset	12.0 mm

**Table 2:** Trial-averaged CAD-constrained rivet-group recovery results in simulation.

Template	MAE-X (mm)	RMSE-X (mm)	MAE-Y (mm)	RMSE-Y (mm)	Mean Minimum Clearance (mm)	Locking Time (s)
1	5.130	5.135	0.762	0.825	0.792	4.58
2	6.655	6.697	0.774	0.821	0.855	10.81
3	5.220	5.225	0.802	0.827	0.921	5.21

**Table 3:** Trial-averaged localization errors for geometry-only and CAD-constrained recovery.

Template	Method	MAE-X	RMSE-X	MAE-Y	RMSE-Y
1	Geometry-only (mm)	5.601	5.989	5.126	6.105
	CAD-constrained (mm)	5.130	5.135	0.762	0.825
	Relative reduction (%)	8.40	14.26	85.14	86.49
2	Geometry-only (mm)	6.656	7.017	5.041	6.105
	CAD-constrained (mm)	6.655	6.697	0.774	0.821
	Relative reduction (%)	0.01	4.56	84.65	86.55
3	Geometry-only (mm)	5.890	6.260	4.762	5.820
	CAD-constrained (mm)	5.220	5.225	0.802	0.827
	Relative reduction (%)	11.38	16.54	83.17	85.79

**Table 4:** Qualitative non-intrusion judgments for Template 1 at different inward offsets.

Inward Boundary Offset (mm)	Trials Judged Non-Intrusive/Total Trials	Non-Intrusive Overlay Rate (%)
12	10/10	100
10	9/10	90
8	9/10	90
6	7/10	70
4	7/10	70

**Table 5:** Member-edge results in simulation, laboratory, and field settings.

Setting	Geometry	Evaluation Records	MAE (mm)	RMSE (mm)	Mean Processing Time (ms)
Simulation	Straight	3 groups; 150/150 valid fits	21.380	22.262	23.14
Simulation	Transition	3 groups; 169/169 valid fits	30.487	35.635	20.36
Laboratory	Straight	1124/1325 valid fits (84.83%)	—	—	20.53
Field	Straight	928/1166 valid fits (79.59%)	—	—	23.37

## Data Availability

The original contributions presented in this study are included in the article. Further inquiries can be directed to the corresponding author.

## Appendix A

**Table A1:** Key algorithmic parameter settings used in the experiments.

Parameter	Description	Value
$h_{\min}$	Minimum protrusion-response threshold	3.0 mm
$k_{\sigma }$	Dispersion coefficient for adaptive protrusion extraction	2.15
$\left[Area_{\min},Area_{\max}\right]$	Candidate connected component area range	$\left[50,3250\right]\;{\mathrm{mm}}^{2}$
$\lambda_{\max}$	Maximum candidate-component aspect ratio	3.3
$\delta_{\rho }$	Huber threshold	4.0 mm
IRLS limit	Maximum IRLS iterations	8
$\tau_{\mathrm{depth}}$	Maximum local valid-cell proportion for edge-candidate extraction	0.2
$\tau_{\mathrm{plane}}$	Reference-surface consistency threshold	25.0 mm
$\tau_{\mathrm{edge}}$	Turning-angle threshold for edge-model selection	40°
Edge RANSAC fit	Iterations; minimum inliers	120; 12
Template search	Minimum observations to start template selection	4
$\sigma_{e}$	RMS-residual scale parameter	18 mm
$N_{\min}^{u}$	Minimum number of valid CAD–observation correspondences	8
Stationary estimate qualification	Minimum number of observations; minimum number of correspondences; minimum matching score	8; 8; 0.65
Template-pose confirmation	Maximum translation update; maximum rotation update; required consecutive qualifying estimates	15 mm; 5°; 3

**Table A2:** Depth-camera specifications and acquisition settings used in the physical experiments.

Parameter	Description	Value
Camera model	—	Intel RealSense D435i
Depth resolution	Width × height	1280 × 720 pixels
Depth frame rate	—	30 fps
Depth field of view	Horizontal × vertical	87° × 58°
Manufacturer-specified operating range	Minimum–maximum range	Approximately 0.3–3.0 m
Manufacturer-specified depth accuracy	At a target distance of 2 m	<2%
